# Volunteering in the front line of the Ukrainian refugee crisis: A brief report from Poland

**DOI:** 10.3389/fpubh.2022.979751

**Published:** 2022-09-29

**Authors:** Jan Domaradzki, Dariusz Walkowiak, Dominika Bazan, Ewa Baum

**Affiliations:** ^1^Department of Social Sciences and Humanities, Poznan University of Medical Sciences, Poznań, Poland; ^2^Department of Organization and Management in Health Care, Poznan University of Medical Sciences, Poznań, Poland; ^3^Department of Promotion and Careers, Poznan University of Medical Sciences, Poznań, Poland

**Keywords:** experiences, motivations, Poland, volunteering, Ukrainian refugees, war refugees

## Abstract

This study aims to analyse the experiences and motivations of volunteers who supported Ukrainian refugees who fled to Poland. Our study was conducted among 196 volunteers in Poznan, Poland who answered questions regarding their experiences with voluntary service during the current refugee crisis and the reasons they became involved in volunteer efforts. We found that although the Polish state had no prior experience in welcoming refugees on a mass scale, the Russo-Ukrainian War has resulted in the emergence of “spontaneous volunteers” who have offered their help and assistance to the flood of refugees sweeping in from its eastern neighbor. We also found that because many Polish citizens believe that helping those in need should not rest solely on the government there are two main motivations to volunteer: a general willingness to help and the willingness to volunteer on behalf of Ukrainian refugees. Finally, while this study highlights that in times of crisis, most people are primarily motivated by humanistic and democratic values, including altruism and public service, emotions also play an important role in influencing people's decision to engage in voluntary service.

## Introduction

From the very first moment Russia invaded Ukraine on 24 February, 2022, the resulting warfare has caused many civilian casualties as well as the destruction of civilian infrastructure. Consequently, thousands of Ukrainians have been forced to flee from their homes and cross the borders of neighboring countries seeking safety, protection, and assistance. According to the United Nations High Commissioner for Refugees (UNHCR) up until 22 of June, 2022, from a total 7,703,857 individual refugees who have crossed the Ukrainian border 4,001,921 have fled to Poland, while 1,230,800 have gone to Russia, 782,742 to Hungary, 659,009 to Romania, 502,716 to Moldova, 510,014 to Slovakia, and 16,655 to Belarus (1). On the other hand, according to the Polish Border Guard this number is even higher and has reached 4,238,000 (2). In contrast, other European countries have registered 2,305,082 total refugees. Thus, because more than 7.5 million Ukrainians have fled the country and approximately one third of its population has been displaced, the UNHCR Commissioner, Filippo Grandi, has argued that while it is “… the fastest growing refugee crisis in Europe since World War II” the Russian invasion has sparked “Europe's largest refugee crisis of the century” (3).

The problem is however, that even before the war started, Poland had received over a million Ukrainian labor immigrants who left their country after Russia's illegal annexation of the Crimean Peninsula in 2014 (4). However, the Polish state had had no previous experience in welcoming refugees. Thus, on the very first day that the war began Polish border crossing checkpoints were filled with the thousands of refugees in a state of distress and anxiety who were waiting in lines of cars reaching 20 kilometers. Most of these refugees consisted of women, children, and those with illnesses – all of whom were hungry, cold, and tired.

As a result of this huge influx of refugees, Ukrainians now constitute 8% of the total Polish population, and one in 10 people in Poland is now Ukrainian. Moreover, the population of most big cities have grown significantly. For example, in Rzeszów the population increased by 35%, in Katowice and Gdansk 25%, in Wroclaw 23%, in Cracow 19%, in Lublin 17%, in Poznan 14%, in Warsaw 13% and in Lodz 11% (5). Consequently, to ease this pressure many refugees are being urged to seek shelter in smaller provincial towns.

Significantly, from the first day of the invasion both the central government, local authorities, and various social actors who organized help and support have adapted an inclusive model of helping the refugees that differs significantly from the one that is common in other European countries and the UNHCR. According to Dr. Grazyna Firlit-Fesnak from the University of Warsaw, this model is characterized by:

- An openness to all Ukrainians fleeing from the war: it was guaranteed that every refugee who had chosen Poland as his or her destination and transit country would be accepted into the country,
- cooperation of the Polish nation and State: from the beginning help for the refuges has been organized both by the public and non-public entities of the State and civil society,
- inclusion and activation of refugees within Polish society and economy, and
- a policy of no mandatory relocation and resettlement (6).

From the moment the war began, Polish officials struggled to help all refugees arriving into the country. Consequently, various measures were imposed by the government, including the organization of web pages with information for the refugees, the establishment of registration points in all major Polish cities (the biggest registration center at the National Stadium was organized in Warsaw), and registration buses which traveled to refugee shelters where Ukrainians could apply for a Polish national identification number (PESEL) that is required by residents in order to access the labor market, health care system, and social benefits. Finally, on 12 March the government adopted a special law for people fleeing Ukraine which guaranteed that they could legally stay in Poland for up to 180 days. It also facilitated the legal employment of refugees without the need for them to obtain a work permit, thus enabling those without a job to register as unemployed, and allowing them access to all public health care services, as well as social and family benefits, and services for those seeking psychological assistance. Additionally, refugees whose stay in the country had been deemed legal were allowed to undertake their business activities on the same terms as Polish citizens. To achieve all of these goals the government has facilitated obtaining the PESEL (7).

Simultaneously, many Polish citizens has engaged in various voluntary services to support the refugees. Consequently, as never before, Poland has seen mass mobilizations of thousands of ordinary people and a true explosion of citizens' commitment to the cause of the refugees. For instance, railway workers helped to organize a mass deployment of refuges throughout Poland; owners of hotels and hostels along the border provided refugees free accommodation; and tens of thousands of volunteers filled their own private vehicles with collected food, medications, thermal blankets, warming supplies, diapers, infant formula, and baby wipes before crossing over the Ukrainian border to pass the supplies to the weary people of Ukraine waiting to enter Poland at the border. Many others offered their time and skills at train stations, refugee centers where they worked at reception points, help in administrative work, Polish translation services, legal advice, psychological support, material supplies, and the organization of transportation services. Moreover, countless Polish citizens have provided the Ukrainians with work and free private accommodation. These services are of special importance since no one was prepared for an international crisis such as this one and the entire Polish system has been seriously overwhelmed as a result. Thus, by engaging in *ad hoc* “crisis management” efforts, volunteers have contributed in helping to alleviate some of the stress placed on the national government.

Although previous research has analyzed volunteers' responses to various crises that have haunted Europe in recent years, including: the migration and refugee crisis in 2015 when a vast number of European citizens engaged in helping migrants and refugees (8–16) and the recent global health crisis when thousands of medical students volunteered to support healthcare professionals' fight against the COVID-19 pandemic (17–21), this study attempted to understand the motivations and experiences of Polish volunteers who supported Ukrainian refugees who fled from Russia's invasion in their homeland into Poland.

## Materials and methods

The study was conducted between 15 March, and 15 May, 2022. Participants were volunteers enrolled in different voluntary services in Poznan, Poland. We used a self-developed questionnaire with 36 questions of various types (yes/no, single and multiple choice, 5-point Likert scale of attitude). It consisted of four sections. The Introduction section gathered volunteers' demographic information regarding their sex, age, nationality, profession, marital status, permanent place of residence, religion, and self-perceived health status. The Materials and methods section included questions regarding the respondents' prior experiences with volunteering. The Results section focussed on respondents' experiences with volunteering during the Ukrainian refugee crisis. The Study limitations section of the questionnaire included questions regarding the main reasons volunteers initially became involved in helping refugees. In this section, respondents were also asked to rate on a scale from 1 (not significant) to 5 (very important) their various reasons for volunteering.

The process of editing and expanding the questionnaire followed the guidelines of the European Statistical System (ESS) (22). First, during an online focus-group meeting three researchers developed a list of important issues on volunteering, which resulted in a questionnaire which was assessed by an additional reviewer. Then, the questionnaire was pre-tested *via* an online platform with five volunteers. Based on our pilot study, two questions were added and four were reformulated. The final version of the questionnaire was approved by the Poznan University of Medical Sciences' Bioethics Committee which also granted its ethics and research governance approval (KB – 228/22).

After receiving final approval, we posted the questionnaire and an invitation to participate in the study on an online platform dedicated to volunteers engaged in helping the Ukrainian refugees in Poznan, Poland. Additionally, a snowball sampling was used as participants were asked to provide referrals to other potential subjects. This in turn helped to recruit additional volunteers. Volunteers were included if they were directly involved in voluntary service with Ukrainian refugees and were eager to participate in the study. Informed consent was obtained from all participants included in the study.

The data collected in the questionnaires were verified and checked for completeness, quality, and consistency and exported into the statistical package JASP (Version 0.16.3). The results are presented as descriptive statistics.

## Results

While the total number of volunteers who offered their help to various NGOs and the local authorities in managing the crisis in refugee centers and reception points in Poznan is unknown, 196 volunteers completed the questionnaire. Our group consisted of 157 females and 39 males (Table 1), with an average age of 33.7 years. While 98% of volunteers were Polish, white-collar workers and students predominated the sample population (49 and 44.9% respectively). The majority of volunteers (54.6%) lived in large city and were either single (41.9%) or married (35.2%). While most of the volunteers were Roman Catholics (60.2%), 17.9% declared themselves to be atheists.

**Table 1:** Socio-demographic characteristics of volunteers.

**Characteristics**	***N* (%)**
*Sex*	
Female	157 (80.1)
Male	39 (19.9)
Median age(years)	28
Mean age(years)	33.7
SD (years)	14.3
Minimum age(years)	16
Maximum age(years)	81
*Nationality*	
Polish	192 (98)
Ukrainian	2 (1)
Other	2 (1)
*Profession*	
Unemployed	3 (1.5)
Pensioner	6 (3.1)
Student	88 (44.9)
Blue collar	3 (1.5)
White collar	96 (49)
*Marital status*	
Single	82 (41.9)
A partnership	31 (15.8)
Married	69 (35.2)
Widowed	3 (1.5)
Divorced	11 (5.6)
*Place of residence*	
Up to 10,000 inhabitants	22 (11.2)
10–50,000 inhabitants	17 (8.7)
51–100,000 inhabitants	14 (7.1)
101–500,000 inhabitants	36 (18.4)
Above 500,000 inhabitants	107 (54.6)
*Religion*	
Roman Catholic	118 (60.2)
Other Christian	1 (0.5)
Believer but not affiliated with any religion	22 (11.2)
Agnostic	14 (7.2)
Atheist	35 (17.9)
Buddhist	2 (1)
Other	2 (1)
*How do you perceive your health?*	
Very good	66 (33.7)
Good	118 (60.2)
Bad	11 (5.6)
Very bad	1 (0.5)

The majority of refugee helpers had various types of volunteer experience before the refugee crisis began (84.2%), and over one-third had been a volunteer more than 10 times (37.2%) (Table 2). Over a half of volunteers who declared themselves as having been engaged in voluntary services in the past were more engaged in occasional, episodic volunteering (55.1%), school volunteering (53.6%), individual volunteering (37.8%), and volunteering in hospitals or hospices (34.7%). At the same time, many respondents declared that apart from voluntary service they were helping those in need in many other ways, including donation of food and clothing organized by various charity collections (53.1%) and donation of one percent of their income tax for charity, as calculated in annual statements, to the selected public benefit organization (43.4%).

**Table 2:** Respondents' previous experience with the voluntary service.

	***N* (%)**
*Have you ever been engaged in voluntary service before?*	
Yes	165 (84.2)
No	31 (15.8)
*How many times have you volunteered before?*	
1	7 (4.3)
2	18 (11)
3–5	46 (28)
6–10	32 (19.5)
>10	61 (37.2)
What volunteer work have you done in the past?*	
Volunteering in response to COVID-19	39 (19.9)
Sports volunteering	41 (20.9)
Volunteering in hospitals, hospices	68 (34.7)
School volunteering	105 (53.6)
Employee volunteering	30 (15.3)
Individual volunteering	74 (37.8)
International volunteering	27 (13.8)
NGO volunteering	35 (17.9)
Volunteering for seniors	21 (10.7)
Other	8 (4.1)
*What type of volunteer work have you done in the past?*	
Action volunteering	108 (55.1)
Long-term volunteering	56 (28.6)
Apart from volunteering do you help in any other way?*	
I donate blood	23 (11.7)
I donate 1 percent of tax for charity	85 (43.4)
I donate food and clothing for collection	104 (53.1)
I text messages for charity	33 (16.8)
No	21 (10.7)

*It was possible to give more than one answer.

The predominant feelings in volunteers evoked by the Russo-Ukrainian War and the influx of refugees were compassion (79.1%), sadness (78.6%) and the willingness to act (75.5%) (Table 3). Simultaneously, many respondents felt anger (66.3%) and fear, either over their loved ones (58.2%), or that the war would also come to Poland (54.6%). For 52% of respondents the most important reason to engage in voluntary services during the refugee crisis was their belief that it is important to help others, while 27.5% believed that it was their moral duty, and 8.2% wanted to be a part of something important. Simultaneously, over a half of respondents did not consult their decision on engaging in voluntary service with anybody (53.6%), while 25% consulted with their friends, 23.5% with their partner, and 14.8% with their parents.

**Table 3:** Voluntary service during the Ukrainian refugee crisis.

	***N* (%)**
*What were your feelings after hearing about the war in Ukraine and the wave*	
of refugees?*	
Sadness	154 (78.6)
Existential anxiety	101 (51.5)
Anxiety	97 (49.5)
Fear that the war will also come to Poland	107 (54.6)
Fear about my own future	97 (49.5)
Fear for loved ones	114 (58.2)
Anger	130 (66.3)
Compassion	155 (79.1)
Willingness to act	148 (75.5)
Nothing, it was irrelevant to me	1 (0.5)
Did you consult your decision on engaging into voluntary service with anybody?*	
Parents	29 (14.8)
Siblings	16 (8.2)
Partner	46 (23.5)
Friends	49 (25)
My fellow students	19 (9.7)
Teacher	1 (0.5)
Neighbors	2 (1)
A Priest	1 (0.5)
Someone else	6 (3.1)
No	105 (53.6)
*What was the main reason to engage in voluntary service during the refugee crisis?*	
I believe it is important to help others	102 (52)
I believe it is my moral duty	54 (27.5)
It gives me the opportunity to pay back for all I have received myself	10 (5.1)
I wanted to be a part of something important	16 (8.2)
To experience the adventure	1 (0.5)
It gives me the opportunity to realize my passion	2 (1)
It is better than sitting at home and studying, or to be bored	4 (2)
To meet new people, make new connections and friends	2 (1)
To put my voluntary participation into my future application documents	1 (0.5)
To gain experience needed in my future profession	2 (1)
To establish new connections that will be useful in the future	0
I was encouraged by a friend who also volunteered	2 (1)
What do you do during voluntary service?*	
I work at the reception point	19 (9.7)
I work at the information point	17 (7.6)
I work at the catering point	21 (10.7)
I help in logistics and transport	36 (18.4)
I organize material collections (sorting, packing and distribution of material supplies)	144 (73.5)
I help with the translation from Ukrainian/Russian/English	10 (5.1)
I work at the Internet and telephone information desk	3 (1.5)
I help in administrative and office work	14 (7.1)
I provide a flat or host refugees at home	32 (16.3)
I offer psychological support	14 (7.1)
I offer legal support	3 (1.5)
I offer material support	103 (52.6)
I offer medical assistance	18 (9.2)
I provide child care for Ukrainian children	25 (12.8)

*It was possible to give more than one answer.

The largest group of refugee helpers organized material collections (73.5%), offered material support (52.6%), helped in logistics and transportation (18.4%), provided a flat or hosted refugees at home (16.3%), or provided child care to Ukrainian children (12.8%).

Interestingly, although many volunteers were concerned that different immunization schedules in Ukraine and overall lower vaccination rates among refugees could pose an epidemiological risk (54.1%), 67.3% were not afraid of the possibility of being infected with COVID-19 (Table 4). At the same time, of those who felt anxious about the possibility of contracting the novel coronavirus, 18.4% believed that in times of war it did not matter and for this reason they felt obliged to engage whatever the risk.

**Table 4:** Volunteers' opinions on the epidemiological risk related to the mass influx of refugees.

	**N(%)**
*Do different immunization schedules (i.e. measles, viral hepatitis) and low*	
*vaccination level among Ukrainians refugees pose epidemiological risk?*	
Yes	106 (54.1)
No	26 (13.2)
I do not know	64 (32.7)
*Does the low vaccination level for COVID-19 among Ukrainians makes you*	
*feel sacred of contracting the various?*	
Yes	24 (12.3)
Yes, but in times of war it does not matter to me	36 (18.4)
No	30 (15.3)
No, because I am vaccinated for COVID-19	80 (40.8)
No, because I believe that currently the pandemic is no longer a risk	22 (11.2)
I do not know	4 (2)

Although the vast majority of respondents declared that the voluntary service among Ukrainian refugees met their expectations (77%) and over 80% declared that it was not as hard as they had expected, still many felt burdened by their lack of knowledge of the Ukrainian language (42.9%), organizational chaos (35.75), the number of duties (16.3%), lack of sleep (11.7%), and long working hours (9.7%). Additionally, 22.4% of volunteers were afraid that their volunteer efforts would not bring tangible results, while 11.7% were anxious about the risk of contracting an infectious disease, including COVID-19 (Table 5).

**Table 5:** Respondents' experience with voluntary service during the Ukrainian refugee crisis.

	***N* (%)**
What is the hardest during your voluntary service?*	
Lack of knowledge of a language	84 (42.9)
The pace of work	10 (5.1)
Duration of work, long working hours	19 (9.7)
The amount of duties	32 (16.3)
Type of performed tasks	10 (5.1)
Organizational chaos	70 (35.7)
Sense of danger related to the risk of contracting infectious disease, including the COVID-19	23 (11.7)
Contacts with the unknown people	18 (9.2)
Contacts with the refugees	3 (1.5)
Lack of sleep	23 (11.7)
Feeling that my work will not bring tangible results	44 (22.4)
Lack of psychological support	14 (7.1)
Are you anxious about anything during your voluntary service?*	
That I will not handle it	26 (13.3)
That I do not poses sufficient skills and competences	37 (18.9)
That the volunteering will negatively affect my studies/work	21 (10.7)
That I will not have time for my family and friends	19 (9.7)
That I will not have time for my personal duties	31 (15.8)
I feared contacts with the strangers	15 (7.6)
That my work input will not bring tangible results	31 (15.8)
I feared the risk of contracting infectious disease, i.e., measles	10 (5.1)
That I can get infected with COVID-19	14 (7.1)
I had no worries	91 (46.4)
*What reactions do you face as a volunteer (including those form your*	
*family, relives, friends)?*	
Positive	151 (77)
Rather positive	40 (20.4)
Indifferent	4 (2.1)
Rather negative	1 (0.5)
Negative	0
*Does voluntary service meet your expectations?*	
Yes	151 (77)
No	6 (3.1)
I do not know	39 (19.9)
What are the benefits of volunteering?*	
Feeling of being needed - increase in self-esteem and motivation	103 (52.6)
Sense of accomplishment and agency	87 (44.4)
The possibility of helping those in need	170 (86.7)
The opportunity to prove yourself	88 (44.9)
Learning practical skills	22 (11.2)
Strengthening of personal and social skills (learning patience, communication, cooperation)	20 (10.2)
(First) work experience or enhancing one's professional skills	59 (30.1)
New relationships, meeting interesting people	12 (6.1)
Increase in self-help in society	47 (24)
The opportunity to spend time usefully	42 (21.4)
Learning about a new culture and language	47 (24)
What are the disadvantages of volunteering?*	
Lack of salary	15 (7.7)
Lack of time for family and friends	39 (19.9)
Lack of time for oneself	50 (25.5)
Negligence in educational or professional duties	50 (25.5)
The risk of contracting infectious disease	28 (14.3)
People's negative reactions	6 (3.1)
I do not see any disadvantages	83 (42.3)
*Was volunteering harder than you expected?*	
Yes	21 (10.7)
No	157 (80.1)
I do not know	18 (9.2)

*It was possible to give more than one answer.

Additionally, although almost half of the volunteers had no worries related to voluntary service among the refugees (46.4%), nearly one-third were concerned over their qualifications which might be inadequate to their responsibilities (18.9%) or that they would not be able to handle the responsibility of volunteering (10.7%). Moreover, some were worried that volunteering would be too time consuming and that they would not have time for personal duties (15.8% apiece).

For all of these reasons some respondents declared that the biggest disadvantage of volunteering was a lack of time, either for educational or professional duties (25.5%), oneself (25.5%), or family and friends (19.9%). At the same time, benefits derived from helping the refugees were stressed much more often, as 86.7% declared that helping others provided them with the realization of the ideal of doing good while 52.6% declared that it helped them grow personally. For 44.9% it was a possibility to prove oneself and 44.4% argued that volunteering gave them a sense of accomplishment and agency.

Finally, more than 97% of volunteers declared having received positive reactions either from their families, fellow colleagues, or friends and very few regretted their decision to volunteer.

Volunteers were also asked to rate their various reasons for volunteering (Table 6). A detailed analysis enabled the identification of four main functions of volunteering among Ukrainian refugees: values, enhancement, career, and social. The values function ranked highest, with a mean of 3.84. Next was the enhancement function (M = 2.54), followed by social (M = 2.25), and career function (M = 2.00).

**Table 6:** Volunteers' motivations.

	**1**	**2**	**3**	**4**	**5**	**Median**	**Mean**
To help others (Value)	0	0	1	42	153	5	4.78
To give something from myself to the community (Value)	7	8	25	59	97	4	4.18
To realize the duty of public service (Value)	49	29	37	36	45	3	3
To participate in something important (Value)	25	31	40	44	56	4	3.38
To do something important and feel proud and fulfilled (Enhancement)	28	37	38	40	53	3	3.27
To pursue the passion of helping others (Enhancement)	30	45	41	36	44	3	3.1
To experience the adventure and to tell in future that I was a part of it (Enhancement)	103	27	29	18	19	1	2.1
To fill free time (Enhancement)	132	22	20	16	6	1	1.68
To get new knowledge and skills (Career)	62	35	35	36	28	3	2.66
To gain professional experience (Career)	114	31	19	16	16	1	1.92
To enhance my professional résumé (Career)	149	24	14	4	5	1	1.43
To make new friends and establish new connections (Social)	76	44	29	35	12	2	2.30
To work with other people (Social)	54	29	42	43	28	3	2.81
To gain recognition of my professors, family and friends (Social)	131	31	15	12	7	1	1.64

Significantly, the three most highly ranked motivations were altruistic/value-driven: the desire to help others (M = 4.78), the desire to give something back to the community (M = 4.18), and the desire to participate in something important (M = 3.38). Simultaneously, while the response “To help others” ranked with the highest score it differed significantly way from every other response. On the other hand, career-related motivations and those appealing to a social dimension of volunteering were among the lowest ranked reasons to volunteer: “To enhance my professional résumé” (M = 1.43), “To gain recognition of my professors, family, and friends” (M = 1.64) and “To fill free time” (M = 1.68).

## Discussion

The Russian invasion of Ukraine has caused a great humanitarian crisis which has led to the displacement of a large number of people who have fled from their homes. At the same time, it has motivated an unprecedented number of Polish citizens to engage in practices to help this vulnerable population. More Polish citizens than ever before have engaged in helping these refugees in many different ways, ranging from working in reception centers, organizing logistics and collections of material supplies, to providing refugees with psychological and legal support. Additionally, they have offered language courses and child care to Ukrainian children. Simultaneously, this study confirms the observation made by other studies that have demonstrated that the most common form of helping refugees is the organization of material collections and support. For example, a recent report conducted by one of the largest Polish NGOs, *Noble Gift*, showed that Poles were the ones primarily involved in providing these refugees with financial support (48%) as well as the organization of food and clothing collections (46 and 34% respectively) (23). Similarly, according to the Center for Public Opinion Research (24) the most popular form of helping these refugees has been the collection of food, clothing, and hygiene products (76%); monetary donations (57%); the provision to refugees of a flat or home (12%); involvement in the organization of various collections (12%); and assistance in official matters (11%) (24).

However, the most important finding from our study is that although volunteers' engagement with migrants and refugees has a long tradition, especially when it comes to third-sector organizations (25), what is new in the current Ukrainian crisis is that it has resulted in a social spurt of volunteering for refugees that has spread throughout the country. Thus, since many refugee helpers from this study believed that helping those in need should not rest solely on the government this “new volunteerism” is best characterized as “spontaneous volunteering” (11, 23, 24, 26–28). Indeed, while according to the Polish Economic Institute at the beginning of the war 70% of Polish citizens engaged in some form of refugee assistance, the majority of that help was *ad hoc*, as 65% of volunteers were helping up to 5 h per week, while only 9% for more than 10 h. Additionally, most of these actions had a local impact (29).

Moreover, because most respondents stressed two main motivations to volunteering: a general willingness to help and willingness to volunteer on behalf of Ukrainian refugees, this research supports other findings that suggest that the majority of the newly engaged volunteers were motivated neither by religious nor political factors. Thus, they were not activists but “ordinary citizens” (23, 29–32).

This research is also in line with findings from other studies that have emphasized that volunteering serves different functions and motivations (33–36). However, while previous studies have suggested that many volunteers nowadays, young people in particular, are less orientated toward value-based volunteering and are more focused on their career and personal growth (37–47), this study demonstrated that in a time of crisis the majority of people have been primarily motivated by altruism and public service, while professional and social motivations for volunteering recorded much lower means. Most volunteers enrolled in this study declared that after hearing about the war in Ukraine and the influx of refugees, they felt a strong need to act and wanted to be a part of the solution. Thus, while they were driven by the idea of doing good and also by the ethical imperative to help those who are disadvantaged, they ultimately stepped forward out of a sense of civic responsibility.

This should come as no surprise, because previous research has shown that although volunteers are aware of the personal benefits derived from volunteering, (i.e., gaining new knowledge and skills or making new contacts that might help them in the future), these are not their primary motivations but are rather additional benefits (40, 44–47). Similarly, most studies conducted among volunteers during the migration and refugee crisis in 2015 emphasized the importance of humanistic and democratic values over other motivations (8–16, 26–28, 48–50). On the other hand, recent studies conducted among volunteers who engaged in the fight against the COVID-19 pandemic demonstrated that in times of a global health crisis people's primary motivations for becoming involved in voluntary service were altruism and the ethical imperative to serve their community and their fellow citizens (17–21, 51).

Also, most volunteers enrolled in this study described helping refugees both as a moral imperative as well as a satisfying experience which made them feel proud and fulfilled. Additionally, many emphasized that volunteering increased their self-esteem, sense of accomplishment, and personal agency. This is in line with findings from other studies which have suggested that new volunteers often distance themselves from controversial activism for refugees and declare humanitarian reasons instead as the reason for their engagement (49, 50). Thus, while most participants from this study felt solidarity with the refugees, humanitarianism was the most often mentioned reason for volunteering (52). However, while other studies have also suggested that the primary motivation for volunteering among the refugees was altruism, our study showed that some volunteers' motivations were also related to personal enhancement, i.e., feeling good about oneself, increasing one's self-esteem, or avoiding feeling guilty (29).

What is equally important to know is that although the war began when the risk of the pandemic was still present and many COVID-19 restrictions were being enforced, most volunteers believed that helping the refugees was a moral duty. Thus, even though at the start of the war there were 5,015,994 confirmed COVID-19 cases and 112,452 deaths in Ukraine and the vaccination rate was very low (36.3%) (53), most respondents felt obliged to engage whatever the risk (20, 54).

However, although most volunteers who responded to the call for assistance were primarily motivated by altruism, still many were not fully aware of the demands that a refugee crisis could place on them, both personally and professionally. Consequently, they found some aspects of their service challenging, difficult, and somewhat frustrating. In particular, respondents were burdened by not knowing the Ukrainian language and culture which hindered their communication with the refugees and made them feel unable to address all the needs of the refugees (55–57). Additionally, as volunteers experienced greater pressure they were frustrated by organizational chaos, work overload, and long working hours which made them feel stressed and uncertain about the results of their work [55, 58]. Thus, it should come as no surprise that the majority of Poles believe that it is the government (30%), European Union (25%), and international humanitarian organizations (17%) that should help, while only 9% declared that everyone should be involved (23). Moreover, a recent study conducted by Szlachetna Paczka found that while 56% of Poles declared that they have been involved in helping the refuges since the war started, the majority believe that Ukrainians should be helped only up until the moment when they will be able to return to their country (69%), while 18% believe that they should be helped and allowed to settle down in Poland permanently (23). Similarly, according to a public research poll conducted in June 2022, the number of Poles who believe that Poland should still accept refugees is slowly decreasing (94% in March vs. 82% in June), while the number of those who are against it is increasing (from 7 to 12% respectively). However, 52% of Poles still declare that they are willing to help the refugees (24).

Finally, our research highlights the importance of emotions as a motivating factor for engaging in voluntary service (15). The majority of volunteers emphasized that feelings of compassion, anger, and a willingness to help have influenced their decision to engage. A similar observation was found by Doidge and Sandri (14) who found that while for most volunteers who helped in an informal refugee camp in Calais, France called the “Jungle”, empathy was the initial motivator for action and also helped to sustain their voluntary service as volunteers and make sense of their emotions through working in the camp. Similarly, a survey conducted by the Center for Public Opinion Research demonstrated that the majority of Poles who engaged in volunteer efforts felt strongly threated by the war (85% in March to 77% in June) (24). On the other hand, another survey showed that the most predominant feeling experienced by those who engaged in helping the Ukrainian refugees was compassion (60%), followed by sadness (45%), uncertainty (44%) and anger (39%) (23). Thus, our study confirms that it was primarily empathy and altruism that motivated Poles to volunteer even before the central government, local authorities, and various NGOs started to help.

## Study limitations

Although to the best of our knowledge this is one of only a few studies conducted on the motivations and experiences of Polish volunteers during the Ukrainian refugee crisis, it has limitations which may have an impact on its generalizability and interpretation. Firstly, because this study utilized a relatively small sample size it reduces the power of the study and increases its margin of error. Consequently, it indicates the need for further investigation. However, it should be noted that many respondents refused to participate in our study due to a lack of time and/or work overload. Secondly, because the study took place when some COVID-19 restrictions still applied it might have hindered the recruitment process and reduced the number of respondents. Thus, it would be desirable to compare the findings to a larger group. Thirdly, since we analyzed responses from volunteers from only one Polish city, this study has a localized impact. Consequently, it would be desirable to compare our findings to other locations in the country. Fourthly, because this research represents solely the opinions of those volunteers who agreed to participate in the study the results cannot therefore be extrapolated to the entire population of volunteers either in Poznan or in Poland as a whole, and more in-depth studies would be required. Another limitation resulted from non-random sampling which prevented an analysis of the socio-demographic, structural, and socio-cultural background of the issues discussed in the study. Finally, as this study is based on the quantitative method only, to better understand volunteers' motivations, needs and lived experiences, further in-depth studies using qualitative methods would be required.

Despite these limitations, however, this study also benefits from some advantages. Most importantly, to the best of our knowledge, no other research on volunteering during the Ukrainian refugee crisis has been done. Thus, as there is a scarcity of previous work on the topic, this research helps to fill a gap in the literature on the attitudes of volunteers toward the Ukrainian refugees and may stimulate further research on the topic. Moreover, because this was a pilot study, it may inspire further research on volunteers' experiences and needs during the current crisis. Finally, as this study enabled the volunteers to share some of their lived experiences it might have a therapeutic value.

## Conclusions

All in all, this study contributes to the existing literature by describing how the current refugee crisis caused by the Russian invasion of Ukraine resulted in the emergence of spontaneous volunteering in Poland. It also shows that when a community is faced with a crisis then people want to be a part of the solution. Moreover, it shows that regardless of a difficult situation and working conditions volunteers are ready to adapt to a crisis to ensure that people who are negatively affected receive the services and support they need. Finally, it demonstrates that although volunteers were motivated by humanistic and democratic values, emotions also played an important role in influencing people's decision to volunteer. At the same time, this research suggests that there is still a need to improve the organizational and internal communication of volunteer organizations.

## Data availability statement

The raw data supporting the conclusions of this article will be made available by the authors, without undue reservation.

## Ethics statement

The study was performed in line with the principles of the Declaration of Helsinki, and, in accordance with local legislation and national guidelines on research involving human subjects, ethical approval and research governance approval was not required. However, since some participants asked for such approval, however, it was obtained from the Poznan University of Medical Sciences Bioethics Committee (KB – 228/22). Written informed consent was also obtained from all respondents enrolled in the study.

## Author contributions

JD, DW, and EB supervised the conceptualization of the study, designed the research questionnaire, and conducted the literature search. DW performed the statistical analyses. JD and DW analyzed and discussed the results and interpreted the data. JD wrote the original draft of the manuscript. All authors collected the data, critically revised, edited the various drafts of the manuscript, and approved the final version of the manuscript.

## Conflict of interest

The authors declare that the research was conducted in the absence of any commercial or financial relationships that could be construed as a potential conflict of interest.

## Publisher's note

All claims expressed in this article are solely those of the authors and do not necessarily represent those of their affiliated organizations, or those of the publisher, the editors and the reviewers. Any product that may be evaluated in this article, or claim that may be made by its manufacturer, is not guaranteed or endorsed by the publisher.

## Abbreviations

HCP: healthcare professionals
PUMS: Poznan University of Medical Sciences
UNHCR: United Nations High Commissioner for Refugees.
